# Advances in Clinical Decision Support Systems: Contributions from the 2023 Literature

**DOI:** 10.1055/s-0044-1800739

**Published:** 2025-04-08

**Authors:** Christoph U. Lehmann, Vignesh Subbiani

**Affiliations:** 1 The University of Texas Southwestern Medical Center, Dallas, Texas, USA; 2 The University of Arizona, Tucson, Arizona, USA

**Keywords:** Medical Informatics, Clinical Decision Support System, Implementation, Expert System

## Abstract

**Objective** : To summarize significant research contributions published in 2023 in the field of clinical decision support (CDS) systems and to select the best papers for the Decision Support section of the International Medical Informatics Association (IMIA) Yearbook 2024.

**Methods** : We refreshed a previous search query for identifying CDS research using Medical Subject Headings (MeSH) terms and related keywords. The query was executed in PubMed in January 2024. Two reviewers reviewed the search results in three stages: title-based triaging, followed by abstract screening, and then full text review. The resulting articles were sent for external review to identity best paper candidates.

**Results** : We retrieved 1948 articles related to CDS, of which four articles were selected as candidates for best papers. The general themes of the final three best papers were (1) improving transfer or discharge timeliness for children in pediatric intensive care units (ICUs), (2) improving acute kidney injury outcomes using medication-targeted interventions, (3) evaluating the safety of medication-related CDS in outpatient settings, and (4) demonstrating potential use cases for CDS in spaceflight missions.

**Conclusion** : Our synopsis highlighted the application of CDS in environments ranging from primary care to pediatric ICUs, and even spaceflight, addressing conditions such as acute kidney injury and bronchiolitis. Ongoing evaluation of the safety and effectiveness of these systems continues to be a central focus of CDS implementation efforts.

## 1. Introduction

Clinical decision support (CDS) systems are central to promoting safe and evidence-based decisions in an increasingly complex healthcare ecosystem. This manuscript represents the synopsis for the Decision Support section of the 2024 International Medical Informatics Association (IMIA) Yearbook. This synopsis supplements the review paper on CDS related to precision medicine, authored by Sulieman et al [ 1 ref to DS survey YB24]. The aim of this synopsis is to summarize significant research in the CDS domain and to select the best papers published in this field in 2023.

## 2. Methods

We used an updated search strategy that we co-developed for the CDS synopsis in the 2023 IMIA Yearbook [ 2 ]. We are including the actual query in Table 1 to support future Yearbook contributors. Similar to the synopsis for 2022, we searched for relevant papers in PubMed® using the revised search strategy. We included journal articles published in 2023 in English with a series of keywords either in the MeSH terms or in the paper's title or abstract. For the MeSH terms, we considered, “Clinical Decision Support”, “Expert systems”, Medical Order Entry Systems”, “Decision Support Techniques”, and “Decision Support Systems, Management”. For the abstracts and title search, we used the terms, “Best Practice Alert”, “Clinical Decision Support”, “Decision Support Techniques”, “Decision Support Systems”, “Expert systems”, “Medication Alert Systems”, and “Computerized Provider Order Entry System”. The final query is shown in Table 1 .

**Table 1.** Search Strategy for CDS Literature Review.

2023[DP] NOT pubstatusaheadofprint AND (journal article[PT]) AND English[LA] AND ( (Clinical Decision Support[MeSH Terms]) OR (Expert systems[Mesh]) OR (Medical Order Entry Systems[Mesh]) OR (Decision Support Techniques[MeSH Terms]) or (Decision Support Systems, Management [MeSH Terms]) OR „Best Practice Alert“[title/abstract] OR „Clinical Decision Support“[title/abstract] OR „Decision Support Techniques“[title/abstract] OR „Decision Support Systems“[title/abstract] OR „Expert systems“[title/abstract] OR „Medication Alert Systems“[title/abstract] OR „Computerized Provider Order Entry System“ [title/abstract] )

We reviewed the query results in three steps: title only review, followed by abstract review, and then full text review as described below. We chose to first triage using a title only review due to the large volume of results from the query (see *Results* section for more details). At this stage, reviewers were blinded to country of origin, institution, or authors. We included any articles that were selected for further review by at least one of section editors (CUL and VS). Following the title only review, we proceeded to screen the remaining articles using their abstracts and again retained papers that were selected by either reviewer. We then individually examined the full text of all remaining articles and held a virtual discussion for each article that was selected by both editors. Through consensus, we identified the candidate best papers. The full IMIA Yearbook editorial committee determined the three finalists and an honorable mention.

## 3. Results

The search query resulted in 1,948 unique references. After the title review, 116 references were included by one or both of the section editors (CUL and VS). After screening the abstracts, 46 articles were identified with six selected by both editors. After full text review, four best paper candidates were selected by consensus ( Table 2 ). Of these, two papers were published in Applied Clinical Informatics and one each in NPJ Microgravity and Nature Communication, respectively. We provide further analyses of the four best paper candidates using the Donabedian's structure-process-outcomes model [ 3 ]. After external review of best paper candidates, the IMIA Yearbook editorial committee selected three candidates as the best papers ( Table 3 ) with the most significant contributions in the field of clinical decision support for 2023. The remaining paper is included in this synopsis as an honorable mention. A summary of the best papers can be found in the appendix of this synopsis.

**Table 2:** Best paper candidates for the Clinical Decision Support Section.

PMID	Title	First Author	Journal	DOI
38092360*	How Safe are Outpatient Electronic Health Records? An Evaluation of Medication-Related Decision Support using the Ambulatory Electronic Health Record Evaluation Tool	Co Z	Appl Clin Inform	10.1055/s-0043-1777107
36792057*	Improving Pediatric Intensive Care Unit Discharge Timeliness of Infants with Bronchiolitis Using Clinical Decision Support	Martin B	Appl Clin Inform	10.1055/a-2036-0337
37344482^	The value of a spaceflight clinical decision support system for earth-independent medical operations	Russell BK	NPJ Microgravity	10.1038/s41526-023-00284-1
37198160*	A randomized clinical trial assessing the effect of automated medication-targeted alerts on acute kidney injury outcomes	Wilson FP	Nat Commun	10.1038/s41467-023-38532-3
*Best paper finalist, ^Honorable Mention				

**Table 3.** Selection of best papers for the 2024 IMIA Yearbook of Medical Informatics for the section Decision Support. The articles are listed in alphabetical order of the first author's last name.

Section Decision Support
• Co Z, Classen DC, Cole JM, Seger DL, Madsen R, Davis T, McGaffigan P, Bates DW. How Safe are Outpatient Electronic Health Records? An Evaluation of Medication-Related Decision Support using the Ambulatory Electronic Health Record Evaluation Tool. Appl Clin Inform. 2023 Oct;14(5):981-991. doi: 10.1055/s-0043-1777107. • Martin B, Mulhern B, Majors M, Rolison E, McCombs T, Smith G, Fisher C, Diaz E, Downen D, Brittan M. Improving Pediatric Intensive Care Unit Discharge Timeliness of Infants with Bronchiolitis Using Clinical Decision Support. Appl Clin Inform. 2023 Mar;14(2):392-399. doi: 10.1055/a-2036-0337. • Wilson FP, Yamamoto Y, Martin M, Coronel-Moreno C, Li F, Cheng C, Aklilu A, Ghazi L, Greenberg JH, Latham S, Melchinger H, Mansour SG, Moledina DG, Parikh CR, Partridge C, Testani JM, Ugwuowo U. A randomized clinical trial assessing the effect of automated medication-targeted alerts on acute kidney injury outcomes. Nat Commun. 2023 May 17;14(1):2826. doi: 10.1038/s41467-023-38532-3.

## 4. Discussion and Outlook

The four best paper candidates were analyzed using various structural, process, and outcome characteristics. Structural aspects include the clinical setting where the CDS was deployed or studied and the type of CDS. The clinical setting of interest included primary care, pediatric intensive care unit (ICU), inpatient hospital setting, and extraterrestrial (i.e., earth-independent) clinical operations during a spaceflight. The type of CDS included a phone alert to notify clinicians about pediatric patients that were ready for transfer from ICU to hospital floor [ 4 ]; medication related electronic alerts [ 5 ]; an active, interruptive alert with targeted information on labs (creatinine levels) and medications related to kidney function [ 6 ]; spaceflight CDS scenarios for diagnostic support, treatment, monitoring, and providing reference information [ 7 ]. Clinical conditions of interest include acute kidney injury, bronchiolitis, life-threatening scenarios ( *e.g.* , events related to ABCDE – airway, breathing, circulation, disability, and exposure), and hypertension.

In terms of process characteristics, we primarily analyzed the nature of CDS evaluation ( *e.g.* , study design; multisite *vs.* single-site studies; provider-facing vs patient-facing CDS; early-stage vs. effectiveness studies). The majority of the CDS systems were clinician-centered, with limited to no involvement of patients or patient-centered factors such as shared-decision making and social drivers of health. Martin *et al.* [ 4 ] conducted a single-site, early-stage, implementation study to evaluate a CDS for identifying likely patient candidates that are ready for transfer from the ICU to the hospital floor. Co *et al.* [ 5 ] conducted a cross-sectional, multi-center study to evaluate medication-related CDS tools in ambulatory setting. The study by Wilson *et al.* [ 6 ] was a prospective, multi-center, open-label, randomized controlled trial to study the effect of an alert-based CDS on rates of discontinuation or cessation of nephrotoxic medications. The paper by Russel *et al.* [ 7 ] was a conceptual overview and demonstration of CDS systems that may be useful for clinical operations in spaceflight missions. Notably, this work took a broader perspective of the value and extent of CDS systems can offer in a resource-constrained and highly controlled environment, while the other three studies were relatively narrow in terms of scope and largely focused on evaluation of rule-based CDS. The outcomes of interest in the four studies included inpatient mortality, progression of acute kidney injury, potential benefits of CDS for a spaceflight mission with an autonomous crew performing medical operations, rates and types of medication orders, length of stay in intensive care units versus hospital, and hospitalization costs.
